# Pan-cancer analysis reveals tumor-associated macrophage communication in the tumor microenvironment

**DOI:** 10.1186/s40164-021-00226-1

**Published:** 2021-05-10

**Authors:** Linbang Wang, Tao He, Jingkun Liu, Jiaojiao Tai, Bing Wang, Zhiyu Chen, Zhengxue Quan

**Affiliations:** 1 Department of Orthopedic Surgery, The First Affiliated Hospital of Chongqing Medical University, Chongqing, 400016 China; 2 Honghui Hospital, Xi’an Jiaotong University, 555 Youyi Dong Road, Beilin, Xi’an, 710054 Shaanxi China; 3 Laboratory of Environmental Monitoring, Shaanxi Province Health Inspection Institution, Xi’an, 710077 Shaanxi China

**Keywords:** Tumor-associated macrophages, Tumor microenvironment, Intercellular communication, FLI1

## Abstract

**Background:**

Tumor-associated macrophages (TAMs) are abundant in the tumor microenvironment (TME). However, their contribution to the immunosuppressive status of the TME remains unclear.

**Methods:**

We integrated single-cell sequencing and transcriptome data from different tumor types to uncover the molecular features of TAMs. In vitro experiments and prospective clinical tests confirmed the results of these analysis.

**Results:**

We first detected intra- and inter-tumoral heterogeneities between TAM subpopulations and their functions, with CD86^+^ TAMs playing a crucial role in tumor progression. Next, we focused on the ligand-receptor interactions between TAMs and tumor cells in different TME phenotypes and discovered that aberrant expressions of six hub genes, including *FLI1*, are involved in this process. A TAM-tumor cell co-culture experiment proved that *FLI1* was involved in tumor cell invasion, and *FLI1* also showed a unique pattern in patients. Finally, TAMs were discovered to communicate with immune and stromal cells.

**Conclusion:**

We determined the role of TAMs in the TME by focusing on their communication pattern with other TME components. Additionally, the screening of hub genes revealed potential therapeutic targets.

**Supplementary Information:**

The online version contains supplementary material available at 10.1186/s40164-021-00226-1.

## Background

Tumor-associated macrophages (TAMs) have been recognized as one of the main immune cell populations in the tumor microenvironment (TME) of most cancers [1]. It has been discovered that TAMs have a complex origin and diverse ontogeny because they originate from both circulating monocyte precursors and tissue-resident macrophages [2]. TAMs are found to have high heterogeneity, for one reason, the accumulation of TAM are originated from both tissue-resident macrophages and blood-borne precursors [3]. It is also reported that functional TAMs contain two polarization states: alternatively activated M2 and classically activated M1 subtypes [4]. There is also a growing consensus that TAMs harbor neither a standard classically activated M1 nor M2 polarization profile, for an example, TAMs in glioma simultaneously express markers of both M1 and M2 activation [5]. Apart from the binary polarization model, a spectral polarization model is also raised for describing the heterogeneity of TAMs, in which TAMs were divided into different subtypes according to different markers and each subtype exert an important role in development of oncology [6].

TAMs are thought to be involved in therapy failure through their role in the TME [7], TAMs have been reported to profoundly involved in the modeling of TME [8], the latter of which is a critical factor responsible for the responsive ratio of immune checkpoint inhibitors (CPIs), also, TAMs inhibit immunotherapy effects in solid tumor by secreting varieties of chemokines, cytokines and enzymes [6], it has been proved that the depletion of TAM restores tumor-infiltrating cytotoxic T cell responses and suppresses tumor growth [3], for an example, the ratio for head neck squamous cancer (HNSC) is 12.2% and 45% for melanoma [9], in melanoma, molecules release by cancer cells, such as CSF-1, can establishing an immunosuppressive TME by recruiting and polarizing pro-tumor M2 macrophages [10], in head and neck squamous cell carcinoma (HNSCC), TAMs are reported to directly facilitate the angiogenesis, invasion, and metastasis of tumor cells [11]. Understanding TAM ontogeny and their interactions with other components of the TME is crucial for clarifying their roles in tumor growth and developing successful cancer therapies [12].

**Fig. 1:**
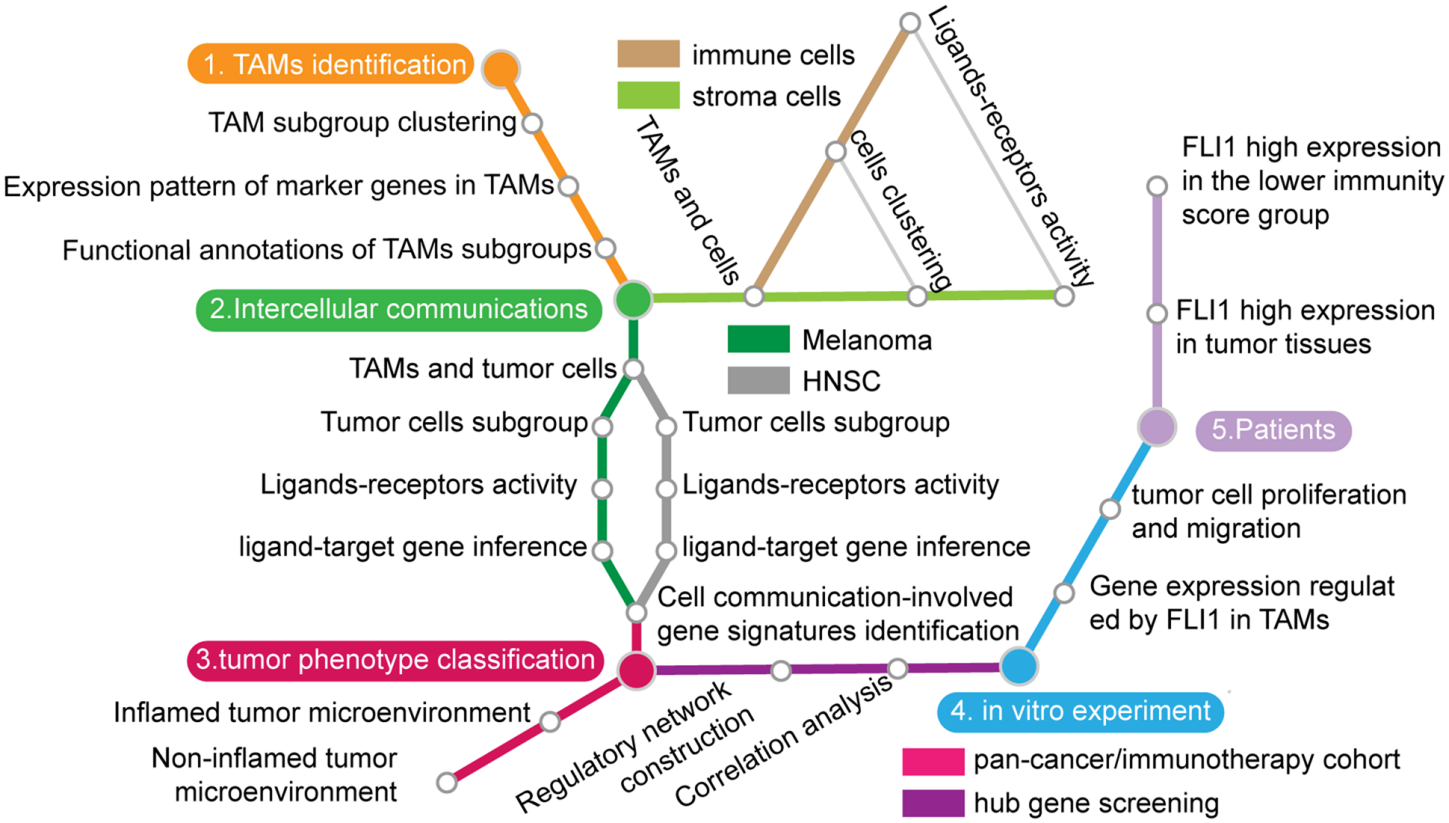
A flow diagram of the study design

To determine the role of TAMs in tumor progression, we selectively analyzed the intercellular communication between TAMs and other components in the TME, including tumor cells, immune cells, and multiple stromal cell types. TME phenotypes, upon pan-cancer analysis, were further divided into different subclusters according to TAM communication patterns. Finally, we identified the transcription factor *FLI1* as one of the hub genes in this process (Fig. 1).

## Results

### Heterogeneity of macrophages and subtype identification

**Fig. 2:**
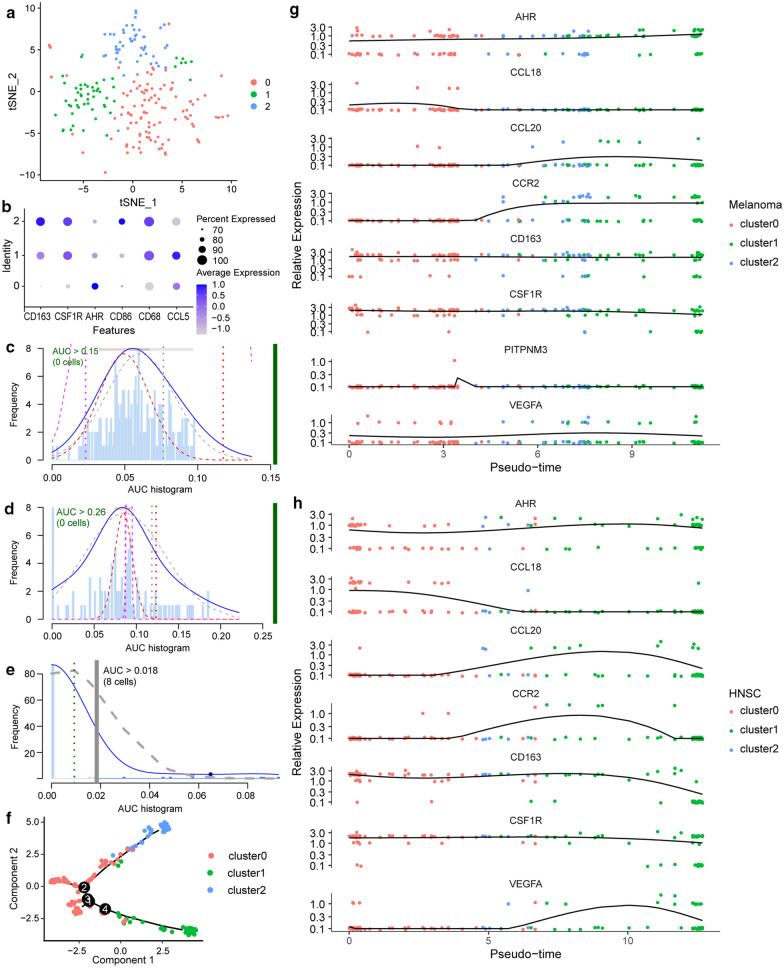
Single-cell RNA-seq analysis revealed molecular characteristics of TAMs in distant tumor types. **a** The t-SNE plot displays infiltrated TAMs. Each dot represents a macrophage and colors represent different clusters of cells. Red, green, and blue one separately represents for cluster 0, 1, and 2. **b** Scatterplot illustrating the expression of TAM markers in each cell clusters. Average expression of each subcluster is represented by color gradient, in which lower expression is represented by lighter color and higher expression is represented by darker color, and the percent of cells is represented by the size of dots. **c**–**e** AUCell R package to identify TAM cluster responses to “Ferroptosis gene sets”. (**c** cluster 2; **d** cluster 1; **e** cluster 0). **f** Construction of TAM differentiation trajectories through pseudotime analysis. **g**, **h** Expression patterns of marker genes enriched in different TAMs clusters in various tumors (**g** melanoma; **h** head neck squamous cancer)

**Fig. 3:**
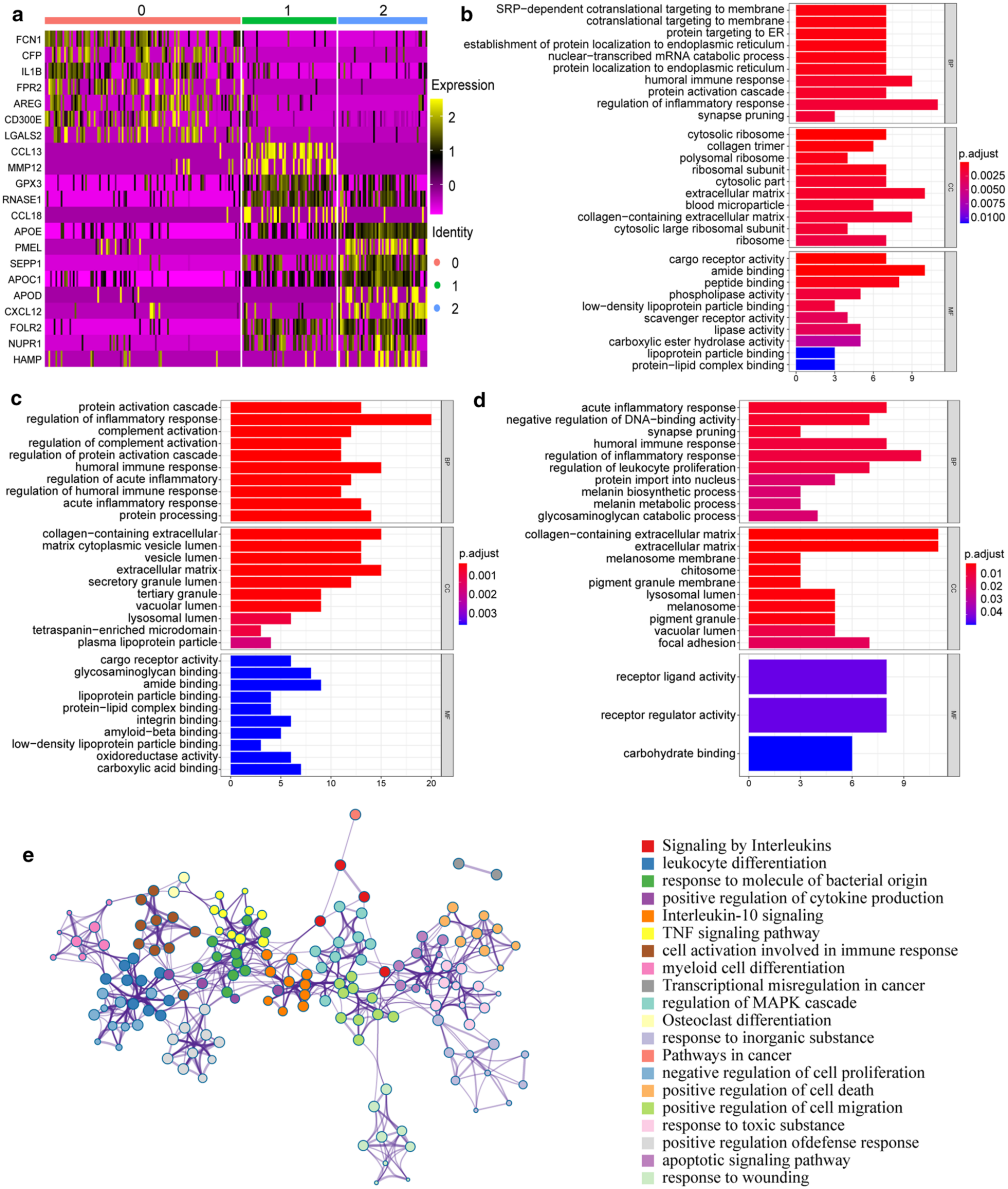
Differentially expressed genes between TAM clusters and their functional annotations. **a** Expression of marker genes in each TAM cluster is shown by heatmap. **b**–**d** The functional enrichment results of newly identified marker genes in different TAM clusters (**b** cluster 2; **c** cluster 1; **d** cluster 0). **e** GO functional annotation network of cell communication gene signatures by Metascape

TAM populations were extracted from different tumors and classified into three subgroups that are illustrated as clusters 2, 1, and 0 (Fig. 2a). First, previously identified TAM markers, including *CD163*, *CSF1R*, *AHR*, *CD86*, and *CCL5*, were used to define the subgroups (Fig. 2b, d). Among them, TAMs in cluster 2 overexpressed *CD86* and thus were named CD86hi TAMs, which displayed a unique signature pattern with the overexpression of *CD163* and *CSF1R*. CD86hi TAMs had low expression of CCL5, which is a feature of M2 macrophages and is predictive of poor patient prognosis [13, 14]. It was newly discovered that CD86hi TAMs have a relatively high expression of *FOLR2*, *CXCL12*, *APOE*, and *CCL18* (Fig. 3a), most of which are immunosuppressive proteins. Functional annotation showed that these cells are involved in cargo receptor activity, secretory granule lumen, and complement activation (Fig. 3b). To be noted, these proteins are also in relatively high levels in HNSC tumors. Cluster 1 TAMs expressed inflammatory markers, such as MMP12, and perform SRP-dependent co-translational protein targeting to the membrane [15] (Fig. 3a, c). Thus, we referred to cluster 1 as “inflammatory TAMs”. Functional annotation results for cluster 0 cells revealed several pathways that are involved in immune regulation, including regulation of leukocyte proliferation and regulation of inflammatory responses. They could also be identified by the high expression of IL1B and FPR2 (Fig. 3a, d). Thus, we named cluster 0 “immunoregulatory-related TAMs”. It should be noted that Dai et al. reported a crucial role of ferroptosis in the macrophages [16], so we examined the level of ferroptosis among different TAM groups. The results showed that CD86hi TAMs have an increased level of ferroptosis (Fig. 2c–e). Pseudotime analysis illustrated that cluster 0 could act as a source of TAMs (Fig. 2f–h), which is confirmed by their high expression of stabilin-1 [17]. TAMs rapidly transition from the monocyte-like state through activation phases [18].

### Subcluster-specific cell–cell communication between tumor cells and macrophages

**Fig. 4:**
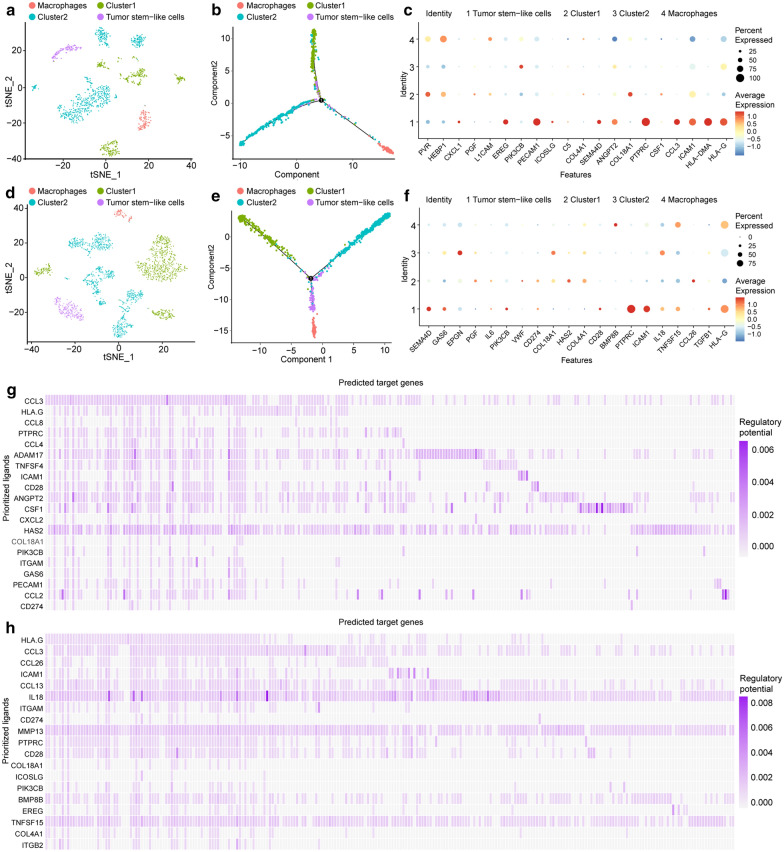
Tumor cell subclusters and TAM-tumor cell communication. **a**, **d** The t-SNE plot displays extracted TAMs and tumor cells in melanoma and HNSC. **b**, **e** Construction of tumor cell differentiation trajectories through pseudotime analysis. **c**, **f** Result of NicheNet’s ligand-receptor activity prediction among TAMs and tumor cells in different tumor types. **g**, **h** Ligand-target gene matrix that denotes the potential regulatory relationships between target genes and ligands among TAMs and all tumor cells

To examine the cell–cell communication among tumor cells and macrophages, we first extracted tumor cells and macrophages from all identified cells for further analysis (Fig. 4a, d). Trajectory analysis was then performed on melanoma and HNSC, and tumor cells were projected onto one root with two branches along with the cell markers in each cluster (Fig. 4b, e). We speculated that the root contains most of the cancer stem cells, whereas most tumor cells were in the branches. Tumor cells within defined branch I in different tumors have upregulation of CTLA4 and CD80, indicating a better response to anti-CTLA4 therapy [19]. However, tumor cells within defined branch II show high expression of PDL1 and PDL2, suggesting a better response to anti-PD1 therapy [20].

TAMs work in cooperation with tumor cells to promote tumor growth. To delineate the cellular communication in tumor tissue, the molecular interactions were modeled using NicheNet, which quantifies the expression level of ligand-receptor pairs and their regulatory genes between tumor cells and TAMs in different tumor types (Fig. 4c, f). A wide range of adhesion molecules, cytokines, and chemokine signaling molecules were identified in interaction pairs, including *IL1B*, *CCL2*, *CCL3*, and *CCL13* (Fig. 4g, h). Interestingly, more than one-third of the genes were shared in different tumors, indicating a homogeneous communication pattern for the TAMs, which was discovered in the pan-cancer analysis. The pathway investigation included genes involved in interleukin-10 (IL-10) signaling, TNF signaling, and regulation of the MAPK cascade (Fig. 3e). Of note, *COL4A1* and *HLA-G* were screened as hub ligands in the TAM-cancer stem cell (CSC) interactions. Additionally, *COL18A1* and *PGF* were screened as tumor cell hub ligands in branches I and II. Altogether, these results showed that the cellular network between tumors and TAMs could foster an anti-inflammatory microenvironment.

### Cell communication gene signatures (CCGS) stratify tumors into TME phenotypes

**Fig. 5:**
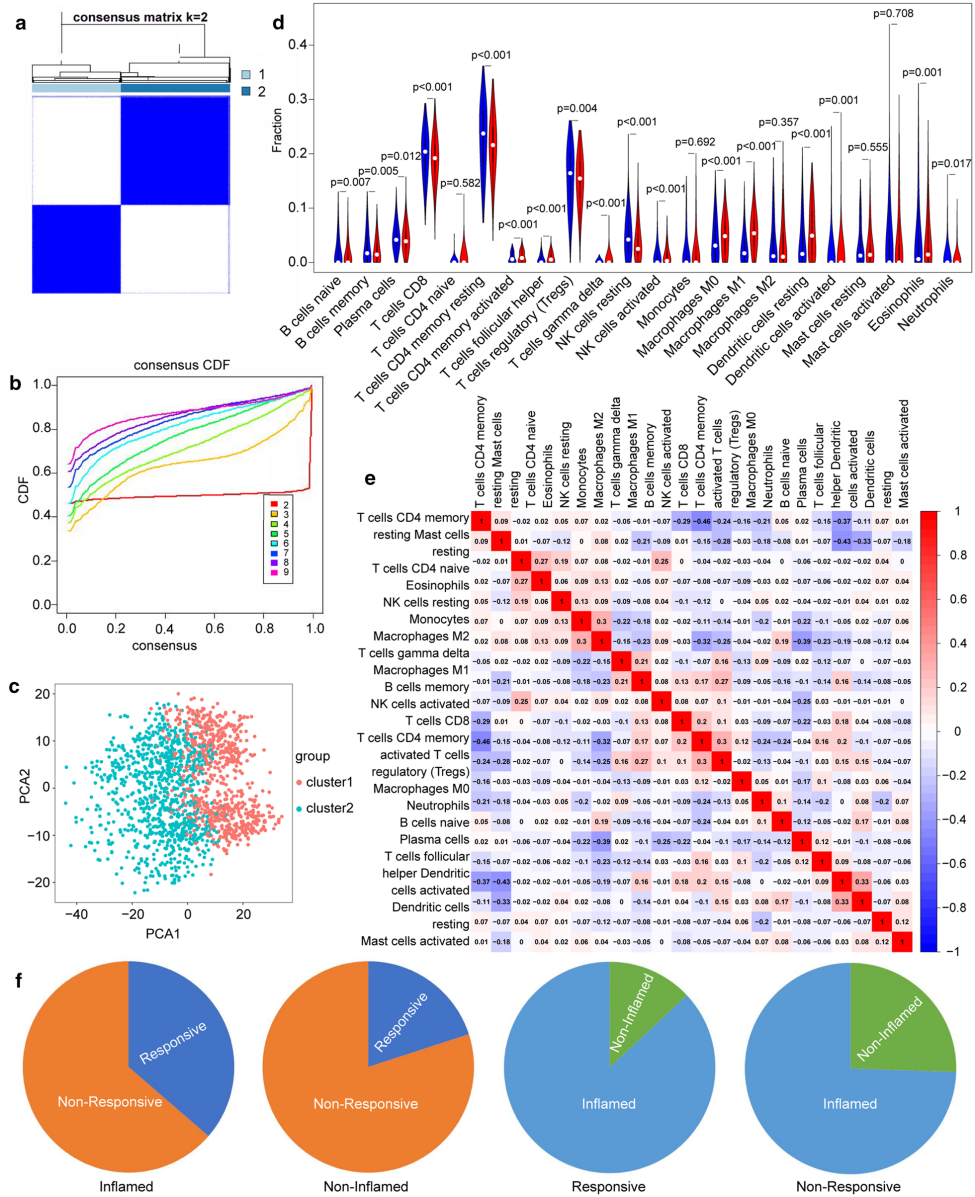
The cell communication gene signature (CCGS)-based clustering of pan-cancer transcriptome samples. **a**, **b** Identification of consensus clusters by 114 CCGSs, the area under the CDF and its relative change with increasing k. The delta area graph shows little variation when K = 2. **c** Reliability analysis of tumor consensus clusters by principal component analysis. **d** The association between tumor phenotype classification and immune cell infiltration. **e** Correlation analysis of the degree of immune cell infiltration. **f** Classified tumor phenotypes are correlated with immunotherapy response

To further explore the biological features of TAM-tumor cell communication in different clinical phenotypes, we focused on the transcriptome data from multiple tumor types. Unsupervised consensus clustering of TNM stage III–IV tumors from 33 different tumor types was performed using the expression of 114 CCGS. The optimal separation was discovered by dividing all the samples into two different subtypes (1: n = 755; 2: n = 933, Fig. 5a, b). Principal component analysis (PCA) results showed perfect separation quality (Fig. 5c). Next, the relative fractions of 22 types of immune cell subpopulations in different clusters were inferred using Xcell and CIBERSORT. We found that cluster 2 showed higher fractions of both innate and adaptive immune cells than those of cluster 1, including multiple types of B cells, activated NK cells, macrophages, and dendritic cells (Fig. 5d, e, Additional file 1: Figure S1F). Thus, cluster 1 was considered to be the non-inflammatory phenotype and cluster 2 subtypes were defined as having an immune-activated phenotype.

To investigate the association between TME phenotypes and the response to immunotherapy, we reproduced our TME phenotype classification using a published melanoma cohort treated with immune CPIs [21] and the same CCGS clustering analysis parameters. As expected, tumors in the inflamed phenotype were the most sensitive to immune CPIs (CR + PR rate: 36.4% in inflamed vs. 20% in non-inflamed, inflamed rate: 87.0% in CR + PR vs. 74.5% in inflamed of these patients under CPI treatment, Fig. 5f).

### Selection and analysis of hub gene significance (GS) genes

**Fig. 6:**
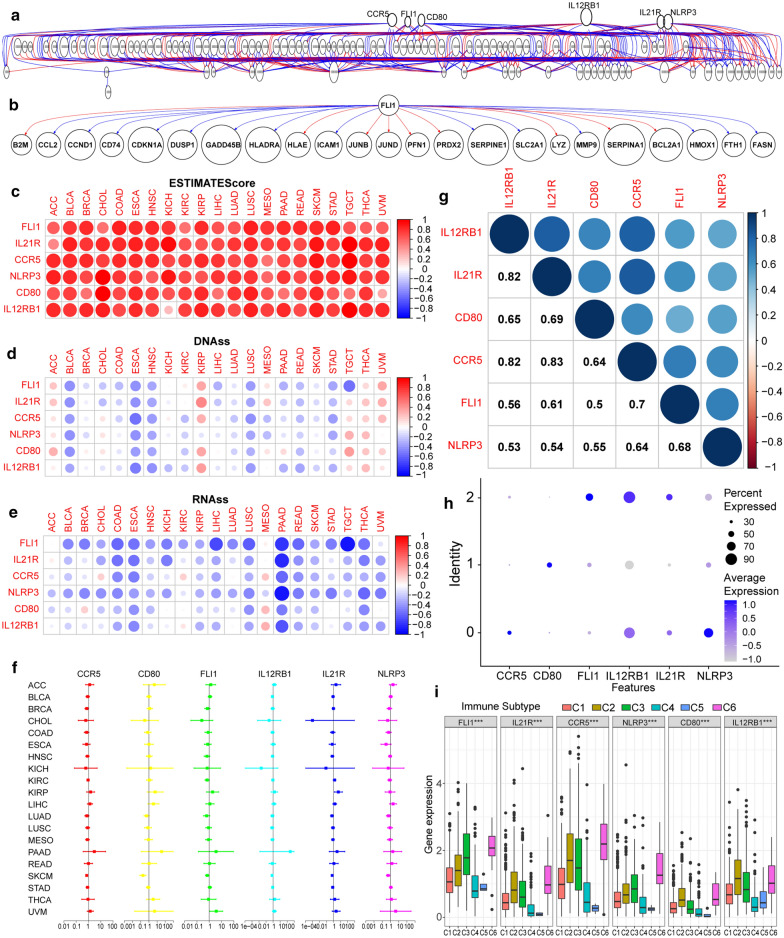
Biological and clinical characteristics of hub genes in multiple tumor types. **a**, **b** GRNInfer was used to construct the cell communication gene signature and Pearson positive regulatory network in pan-cancer for hub genes screening. **c**–**e** Relationship between the estimate, the DNA and RNA stemness scores and hub gene expression levels in multiple tumors. **f** The Cox regression analysis, used to determine the association between hub gene expression and patient prognosis on a pan-cancer scale, illustrated in a forest map. **g** Correlation analysis of hub gene expression. **h** Scatterplot illustrating the expression of hub genes in each cell clusters. **i** The expressions of hub genes in different immune subtypes, shown in a box plot

**Fig. 7:**
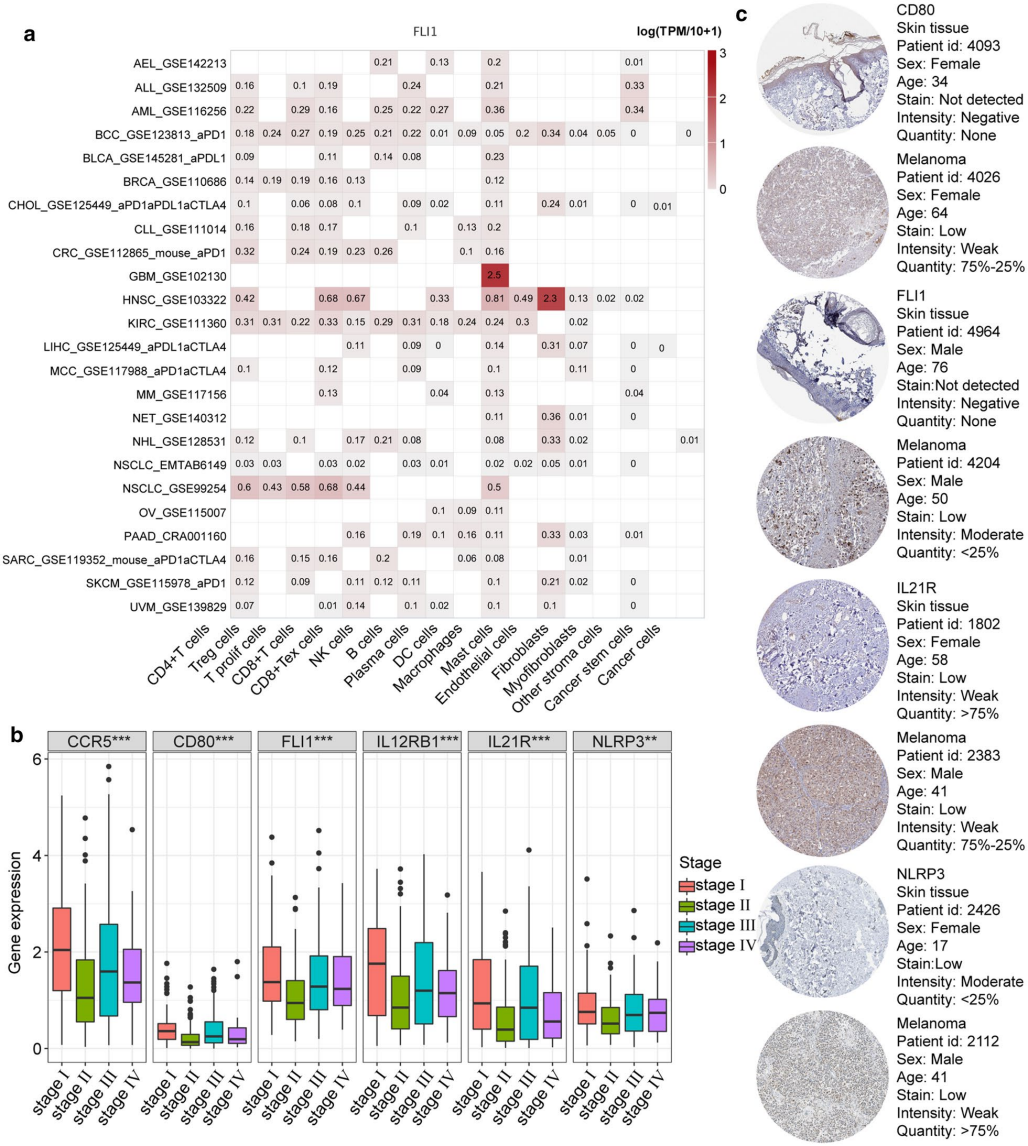
Expression features of *FLI1*. **a** *FLI1* expression at the single cell level in pan-cancer analysis. **b** *FLI1* expression levels in different TNM stages of melanoma. **c** Immunohistochemical staining for CD80, FLI1, IL21R, and NLRP3 between melanoma and normal skin tissues

Based on the CCGS gene set screening, we further constructed the gene regulatory network using GRNInfer (Fig. 6a, b). Out of 114 CCGS genes, six hub genes were identified, including *CCR5*, *CD80*, *FLI1*, *IL12RB1*, *IL21R*, and *NLRP3* (Fig. 6a). Next, the immune-score and the gene expression-based stemness index for the hub genes were evaluated (Fig. 6c–e). The degree of immune cell infiltration was reflected by the estimate scores, which had significant correlation with all genes (Fig. 6c). In addition, the DNA stemness score (DNAss) had a significant positive correlation with adenoid cystic carcinoma, kidney renal papillary cell carcinoma, thyroid cancer, and metastatic uveal melanoma (Fig. 6d). For the RNA stemness score (RNAss) there was a significant positive correlation with mesothelioma (Fig. 6e). We observed the same expression pattern of hub genes in different clusters of TAMs. Interestingly, *FLI1* was found to be highly expressed in CD86+ TAMs (Fig. 6h). Finally, we used Cox regression to determine if gene expression was related to patient survival. The forest plot results demonstrated that the hub genes had a negative prognostic influence on most cancers, except for kidney renal papillary cell carcinoma, pancreatic adenocarcinoma, and adenoid cystic carcinoma (Fig. 6f). To be noted, we utilized the results reported by Li et al. [21], in which, the TME was classified into six different immune subtypes, including C1: wound healing, C2: IFN-γdominant, C3: inflammatory, C4: lymphocyte depleted, C5: immunologically quiet, and C6: TGF-βdominant. As expected, all hub genes were found to be expressed at higher levels in the C6 subtype, which is also known for having high TAM infiltration and the poorest prognosis (Fig. 6i). Through protein expression scanning by the Human Protein Atlas, overexpression of *FLI1*, *CD28*, *CD80*, and *IL21R* was discovered in melanoma (Fig. 7c).

### *FLI1* regulates gene expression in TAMs

**Fig. 8:**
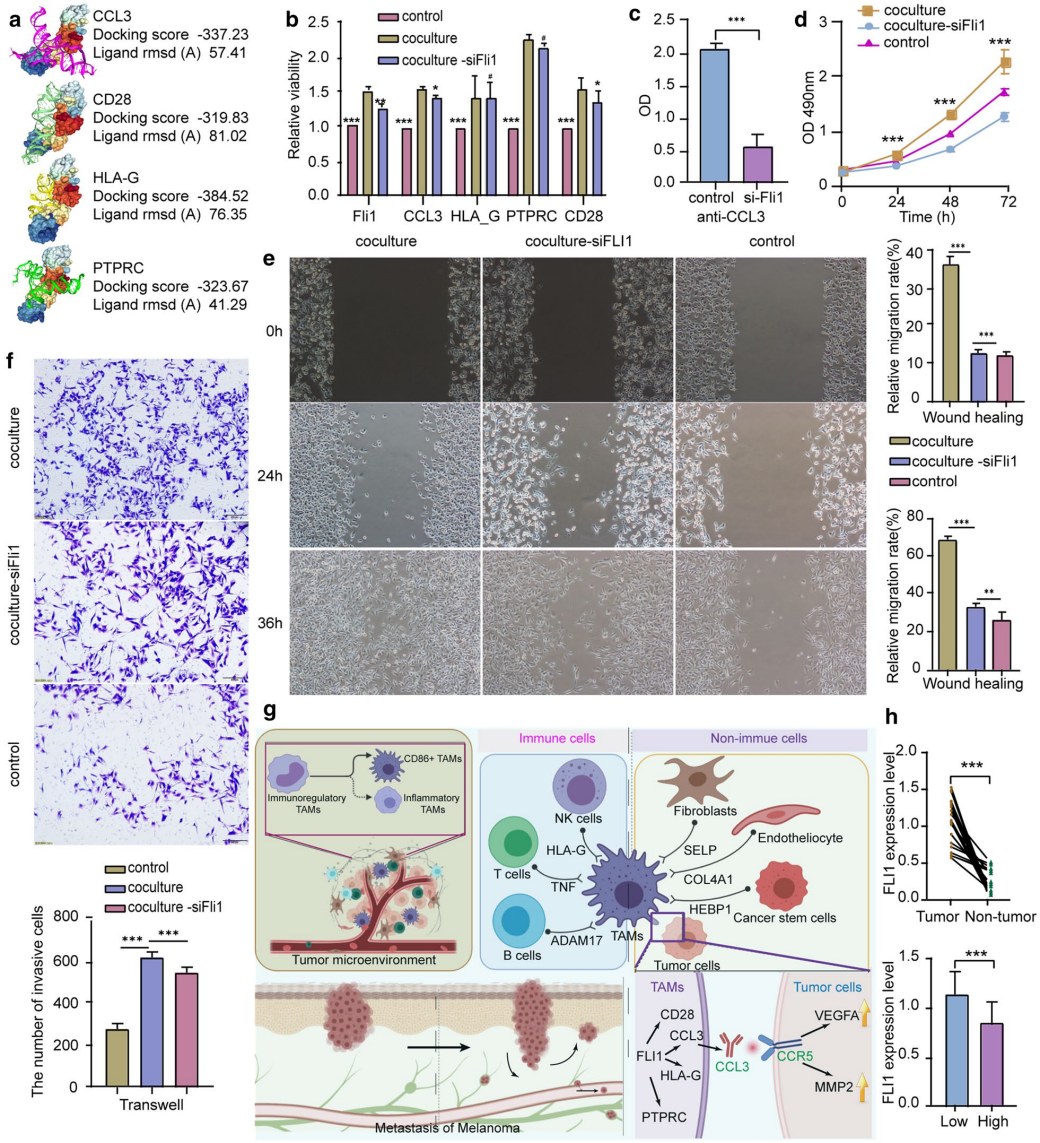
Immunohistochemical staining for CD80, FLI1, and IL21R between melanoma and normal skin tissues. **a** FLI1 binding sites and downstream targets, including CCL3, CD28, HLA-G, and PTPRC were identified. Docking quality of the model was confirmed by HDock. **b** FLI1 expression and its targets after FLI1 knockdown, under co-culture conditions. **c** The protein levels of secreted CCL3 following FLI1 knockdown. **d** Melanoma cell viability when co-cultured with TAMs; detected by a CCK-8 assay. **e** The migration of melanoma cells after co-culturing with TAMs; detected by a wound healing assay. **f** The invasion of melanoma cells after co-culturing with TAMs, detected by a transwell invasion assay. **g** Schematic diagram of TAMs’ role in the TME. **h** Top: FLI1 expression levels in melanoma and normal tissue; bottom: FLI1 expression levels in higher and lower immunity groups. “High” stands for the higher immunity group and “low” stands for lower immunity group

Among all the hub genes, *FLI1* is the only transcription factor. Thus, we selected *FLI1* for further investigation. To explore the pan-cancer expression pattern of *FLI1*, we used TISCH at the single-cell level. *FLI1* had relatively high expression in the macrophage/monocyte population (Fig. 7a). In order to further validate the expression of FLI1 in TAMs, we analysis the transcriptome data of TAMs extracted from different groups of tumor tissues before and after chemotherapy. The result shows that the expression of FLI1 is confirmed to expressed in every sample, and a higher expression of FLI1 is found in the sample of after chemotherapy group (Additional file 1: Figure S1G). We also visualized the expression of *FLI1* in different stages of melanoma (Fig. 7b). Next, we focused on the possible molecular mechanism of *FLI1* in TAMs by using the position weight matrix (PWM) method and molecular docking analysis to screen for DNA binding sites in downstream genes (Fig. 8a). The results showed that the DNA-binding subunit of the FLI1 protein could bind with a high degree of precision with *CCL3*, *CD28*, *HLA-G*, and *PTPRC* genes. Further qRT-PCR analysis showed that the knockdown of *FLI1* by siRNA in M2-like TAMs had a similar downregulating effect on these four downstream genes (Fig. 8b, Additional file 1: Figure S1A). To detect this regulating effect of *FLI1* at the protein level, a *CCL3* ELISA was performed on supernatants of macrophages and revealed that CCL3 was significantly decreased (Fig. 8c).

### TAMs promote tumor cell proliferation, invasion, and migration through *FLI1*

To determine whether *FLI1* expression is involved in TAM-mediated CCL3 secretion or TAM-induced tumor metastasis, si-FLI1 treated RAW264.7 cells and Human Bone Marrow-derived macrophages were co-cultured with B16 melanoma cells and humanized melanoma cells A-375. CCK-8 results showed that tumor cell viability of both types significantly increased after co-culturing with TAMs (Fig. 8d). The migration and invasion of melanoma cells were measured using wound healing and transwell assays. The results showed that the number of migratory and invasive cells was significantly lower in the presence of siFLI1 compared with the co-culture group (Fig. 8e, f, Additional file 1: Figure S1D, E). In order to further investigate the correlation between FLI1 and CCL3 that caused the migration of melanoma cells, we conducted a rescue experiment by adding exogenous CCL3 into the co-culture system, the result shows that exogenous CCL3 reverse the inhibit effect of tumor cell migration that siFLI1 conducted (Additional file 1: Figure S1C–E). These findings indicate that TAMs may promote tumor development through *FLI1*.

### Higher expression of *FLI1* in tumors from patients with low immunity scores

The expression levels of *FLI1* in different groups of patients were detected by qRT-PCR (Table 1). It was found that the expression of FLI1 in tumor tissue (23 cases) and the lower immunity score group (12 cases) were dramatically higher than that in adjacent skin tissue (23 cases) and the higher immunity score group (11 cases), respectively (Fig. 8h).

**Table 1:** Results of immune function quality of host in different groups

Item	Patient 1	Patient 2	Patient 3	Patient 4	Patient 5	Patient 6	Patient 7	Patient 8	Patient 9	Patient 10	Patient 11	Patient 12
ZLBXB-FZ1	16.98	22.61	17.63	25.23	21.52	21.41	17.68	22.34	25.52	21.75	18.74	38.12
CD3+	69.73	85.58	79.45	80.23	72.56	68.23	87.66	82.77	75.69	75.27	84.53	64.75
CD3+CD4+CD8−	36.12	58.29	55.61	47.41	38.89	34.15	56.12	51.92	46.82	31.20	56.21	46.31
CD3+CD4−CD8+	29.27	26.41	28.63	32.61	32.77	29.53	26.31	26.23	31.63	34.22	23.23	19.43
CD4+CD8+	1.01	1.22	0.46	1.72	1.09	0.73	1.87	0.57	1.80	0.80	1.68	0.21
CD4−CD8−	6.07	4.84	2.43	2.81	10.65	5.01	4.16	2.69	3.88	10.93	4.63	2.65
CD3−CD19+	12.71	3.82	3.77	3.10	3.98	15.74	3.08	4.52	2.98	4.56	3.84	14.23
CD3−CD16/56+	15.82	14.26	13.91	13.15	22.91	14.47	13.72	10.23	17.22	21.71	13.81	13.51
CD3+CD16/56+	6.23	7.92	5.61	3.88	6.98	6.23	7.68	5.88	3.62	7.30	7.41	0.88
High or low flag	L	L	L	L	L	L	L	L	L	L	L	H

Item	Patient 13	Patient 14	Patient 15	Patient 16	Patient 17	Patient 18	Patient 19	Patient 20	Patient 21	Patient 22	Patient 23	Reference range
ZLBXB-FZ1	19.17	42.73	34.01	38.54	22.76	37.18	16.99	42.66	38.41	32.31	22.54	27.90–37.30
CD3+	58.26	68.27	66.09	75.29	73.90	69.12	60.31	66.86	70.31	76.44	73.52	26.00–76.80
CD3+CD4+CD8−	49.27	42.98	46.58	45.87	46.88	46.21	44.32	41.42	40.21	48.64	44.63	30.00–46.00
CD3+CD4−CD8+	16.72	22.79	26.88	24.76	26.18	20.34	14.11	29.31	25.87	27.17	24.61	19.20–33.60
CD4+CD8+	0.77	2.81	0.66	0.62	0.62	0.80	0.77	2.80	0.60	0.19	0.77	0.00–2.00
CD4−CD8−	1.54	1.62	1.45	2.87	1.34	3.21	1.45	1.29	1.33	3.21	2.53	0.00–12.00
CD3−CD19+	27.81	14.29	26.41	15.31	15.23	19.26	26.42	13.14	28.61	14.31	12.95	8.50–14.50
CD3−CD16/56+	14.89	14.61	9.31	9.18	9.14	10.41	13.21	15.34	7.63	7.58	12.13	9.50–23.50
CD3+CD16/56+	1.35	4.78	6.79	3.89	2.11	0.88	2.11	4.77	6.23	3.22	1.51	–
High or low flag	H	H	H	H	H	H	H	H	H	H	H	–

### TAM and immune cell interactions

**Fig. 9:**
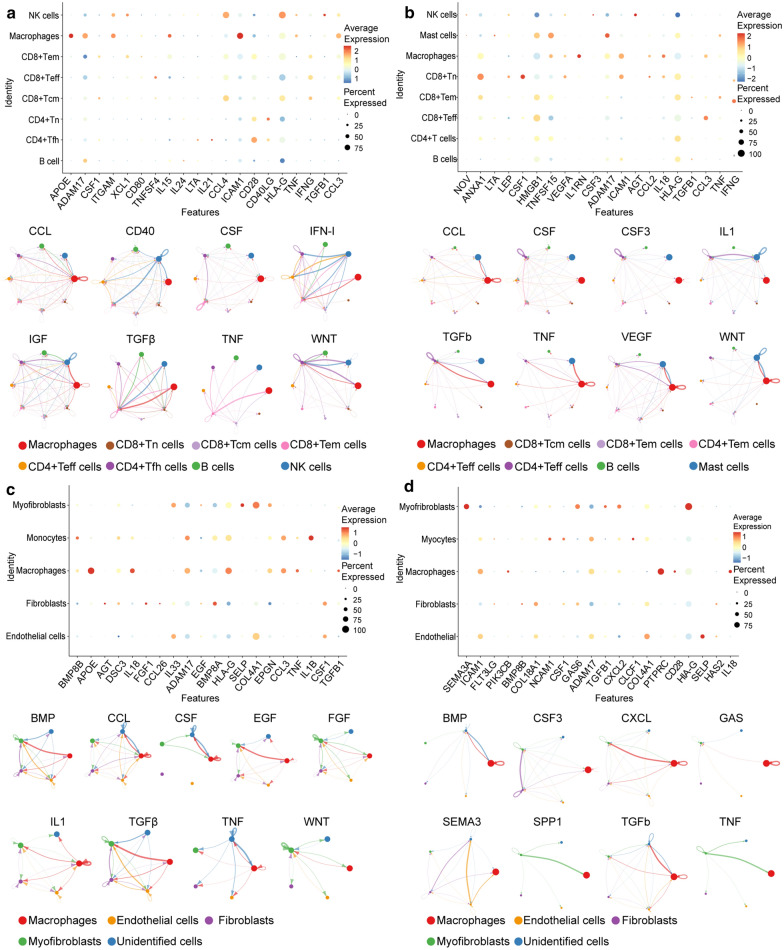
Top: results from NicheNet’s ligand-receptor activity prediction among TAMs and immune/stromal cells in different types of tumors. Bottom: result of Cellchat’s predictions of the signaling pathways involved in intercellular communication (**a**), TAM-immune cell communication in melanoma (**b**), TAM-immune cell communication in HNSC (**c**), TAM-stromal cell communication in melanoma (**d**), TAM-stromal cell communication in HNSC

To examine cell–cell communication between TAMs and each subtype of immune cells, we used both NicheNetr and CellChat to infer possible unbiased receptor-ligand interactions between cells in each sample. First, all of the infiltrated immune cells were identified and extracted, including TAMs, NK cells, mast cells, T cells, and B cells. T cells were further divided into central memory T cells (CD8+ Tcm), effector memory T cells (CD8+ TEM), naïve T cells (CD4+ TN), effector T cells (CD4+ Teff), and follicular helper T cells (CD4+ Tfh), according to the expression of CD4, CD8, CD45RA, and other co-stimulatory molecules [22–24]. The results showed that *ADAM17*, *HLA-G*, and *TNF* are found to be involved in cell communication between TAMs and B cells, NK cells, and T cells, respectively (Fig. 9a, b top). CellChat results revealed that signaling pathways, including TGF-β, WNT, and CCL, are highly involved in the communication between immune cells (Fig. 9a, b bottom).

### TAM and stromal cell interactions

Using a similar protocol, we detected cell–cell communication between TAMs and tumor-infiltrating stromal cells. First, stromal cells, including myofibroblasts, fibroblasts, and endothelial cells, were identified. The results showed that fibroblasts expressed high levels of BMP8A and endothelial cells expressed high levels of SELP and COL4A1, which interact with TAMs (Fig. 9c, d top). CellChat results showed that singling pathways, including BMP, CSF, and TNF, are highly involved in immune cell communication (Fig. 9c, d bottom).

## Discussion

In most tumor types, TAMs are a critical step in the process of tissue remodeling, angiogenesis, and suppression of tumor adaptive immunity [25]. In breast cancer in vivo models, the regulation of TAMs via chemotaxis has been proven to be an effective strategy for anti-tumorigenic therapeutic agents [26]. TAM-focused therapies are potentially powerful when synergize with both chemotherapy and immunotherapy [27]. In our study of melanoma and HNSC patients, large-scale single-cell RNA (scRNA)-seq data were re-analyzed to dissect the effect of TAMs in the TME. First, three cell clusters of TAMs were identified. They showed drastically different functional roles. Among them, CD86hi macrophages displayed a specific gene expression pattern that was similar to M2 macrophages. As reported by Chen et al., the presence of CD86^+^ TAMs were significantly correlated with aggressive tumor phenotypes and it has been considered as a promising hepatocellular carcinoma prognostic biomarker, as a costimulatory molecule [28], CD86 is reported to be curative effect evaluation factor under chemotherapy treatment in colon cancer [29]. Of note, CD86hi macrophages are in the complement cascade, which is crucial for the immune response to cancer and is activated by tumor cell and TAM collaboration [30]. In clear-cell renal cell carcinoma (ccRCC), samples containing more C1q-producing TAM infiltrates exhibited immune suppression in the TME [31]. It has also been proposed that the antitumor efficacy of anti-PD-1/PD-L1 CPIs could be enhanced by complement system inhibition [32]. Thus, we hypothesized that CD86hi TAMs are the major TAM subset that contributes to immune therapy resistance (Fig. 8g).

Cancer stem cells (CSCs) are a subset of tumor cells with stem-like properties and are vital in tumor initiation and progression. They reside in the cellular niche, which comprises numerous cell types, including TAMs, which are believed to provide a unique microenvironment for CSC protection while promoting their functions. Recent advances have revealed complex cross talk between TAMs and CSCs [33]. scRNA-seq has provided us with deeper insight into the intra-tumoral cellular heterogeneity. We extracted CSC cell groups from different tumors and focused on intercellular communication across multiple tumor subgroups, including CSCs and TAMs. Extracellular protein signals and their gene regulatory networks that are mostly involved [27], as well as the complexity and conservation of crosstalk between different TME cell components were revealed. Among them, human leukocyte antigen-G (HLA-G) is a cell-surface antigen that is important for carcinogenesis and immune tolerance. It has been reported that cervical CSCs secrete high levels of HLA-G and are suggested to be potent immune modulators that favor cervical cancer cell survival. By generating cell-like properties from induced pluripotent stem cells, we provide a new approach for insight into cervical cancer stem cells and the identification of new oncogenic targets [34].

The effect of TAM-tumor cell communication on the overall characteristics of the TME is not fully understood, as most studies have focused on a single regulator or TME cell type. We have discovered two distinct TME phenotypes with distinct TAM-tumor cell communication patterns, including an inflammatory and non-inflammatory TME phenotype, which may strengthen the understanding of TAM-induced immune responses. Molecular classification of TME phenotypes provides deeper insight into the immunotherapeutic heterogeneity and mechanism of immunotherapeutic resistance. In inflamed, non-responsive tumors, the MAPK-and TNF signaling pathways are upregulated. Interestingly, it has also been demonstrated that TME inflammation is related to enhanced cytokine IL-10 expression, suggesting its dual role in antitumor immunity. Increasing evidence has shown that IL-10 blockade and activation enhance antitumor immunity in different situations [35]. These abnormally regulated pathways may be potential targets for improved immunotherapy in the future. The success of adoptive cell therapy cannot be reproduced in solid tumors mostly due to the influence of their microenvironment. Therefore, an easier therapeutic approach may be to target the inflamed TME selectively, rather than targeting the solid tumor.

Relatively small numbers of gene expression signatures have utility for predicting clinical therapeutic responses to antitumor agents [36]. In our case, GRNinfer identified six CCGS genes as hub genes. Expression analysis showed that hub genes were expressed at a relatively low level in stage II and bounced back in stage III, suggesting TAM restraint followed by a strengthening process. Further analysis revealed diversity between the expression of hub genes and overall patient survival with different tumor types. Also, hub gene expression levels are highly heterogeneous between different subclusters of TAMs. However, a relatively robust relationship was found between hub gene expression and the immune environment in different types of tumors. Interestingly, all hub genes were found to be highly expressed in the C6 immune subtype of tumors, suggesting that TGF-β may be involved [37].

Abnormal expression of *FLI1* in macrophages is involved in multiple immune diseases, with its low expression playing an important role in scleroderma pathogenesis and the development of tissue fibrosis [38]. Overexpression of *FLI1* in macrophages increases LPS-activated IL-27 production [39]. We have also found a higher expression of FLI1 in the sample of after chemotherapy group, which is an interesting discovery since TAMs are known to contribute to the survival of the tumor cells under the chemotherapies by offering advantageous survival signals to the tumor cells induce tumor metastasis [40]. Our co-culture experiment showed that *FLI1* is involved in CCL3 secretion of TAMs and promotes tumor cell invasion. It has also been reported that in esophageal squamous cell carcinoma, TAMs express and secrete CCL3, which contributes to tumor migration, invasion, and angiogenesis by binding CCR5 and activating the PI3K/Akt pathway [41]. Combined with our TAM sub-clustering results, these results indicated that CD86+ TAMs could secrete CCL3 and play a positive role in tumor development. Therefore, CD86+ TAMs are a potentially valuable target for cancer immunotherapy.

In our study, complex signaling was observed between the macrophage populations and immune cells in the microenvironment. However, the interactions between TAMs and other immune cells among different tumor types are homogeneous. TAMs suppress antitumor, inflammatory, and even adaptive immune responses by secreting immunosuppressive factors such as TGF-b, 2,3-dioxygenase, and PGE2 [4]. For instance, TAM secretion of IL-10 inhibits local production of IL-12, thus weakening the Th1 response and NK cell cytotoxicity [42]. We have found that *ADAM17*, *HLA-G*, and *TNF* are involved in cell communication between TAMs and B cells, NK cells, and T cells, respectively. Among them, *ADAM17* is expressed by TAMs in breast cancers and is involved in the regulation of pro-inflammatory mediators, including cytokines, such as cyclooxygenase-2 [43]. Genetic deletion of *ADAM17* in leukocytes leads to later onset of breast cancer growth [44], while HLA-G is produced by tumor-infiltrating microglia in most glioblastomas [45].

Increasing evidence has shown significant supporting roles of non-malignant stromal cells in tumor progression [46]. We used a single-cell transcriptomic approach to identify the stromal components and their interactions with TAMs. *SELP* and *COL4A1* are involved in TAM communication with fibroblasts and endothelial cells. *SELP* may promote macrophage migration and fibroblast collagen production [47], and increased *SELP* mRNA expression correlates with poor overall survival in advanced epithelial ovarian cancer [48]. *COL4A1*, a subunit of type XIII collagen, is a crucial gene for cancer cell migration and invasion [49].

## Conclusions

In conclusion, we identified 3 subclusters of TAMs that exhibited heterogeneous biological features, the patients in a pan-cancer scale were divided into two groups according to the difference of TAMs-tumor cells communication patterns. FLI1 are considered as effective diagnostic markers and potential targets for therapy. Finally, The TAMs-immune cells communications and TAMs-stroma cells communications are revealed.

## Materials and methods

### Pan-cancer transcriptome samples and immunotherapy-treated clinical cohorts

RNA sequencing data across 33 tumor types, along with their updated clinical data, were downloaded from The Cancer Genome Atlas (TCGA) (https://portal.gdc.cancer.gov/). Transcriptome data from clinical tumor samples treated with anti-PD1 and anti-CTLA4 were downloaded from the GEO database (GSE91061). The TAMs were identified according to the original annotations of the gene expression matrixs. Transcriptome data of extracted TAMs from different groups of tumor tissues before and after chemotherapy were downloaded from the GEO database (GSE134600). To discover the epigenetic features of TAMs and their communications patterns with other TME cells, we integrated two single-cell RNA sequencing datasets, including an HNSC cohort (GEO accession number: GSE103322) and a melanoma cohort (GSE72056).

### Cell-type and intercellular communication analysis of RNA-seq data

The scater R package was used to perform quality control of single-cell RNA-seq data. The scimpute R and scran R packages were used for imputation and normalization. To be specific, First, the standard preprocess (log-normalization) was conducted to the two scRNA dataset based on a variance stabilizing transformation (“vst”) in order to identify their variable features individually. Next, anchors between the individual datasets were found using the FindIntegrationAnchors function, which takes both of our Seurat objects as input. Then these anchors were passed to the IntegrateData function and returned as a Seurat object and were applied for downstream analysis. Tumor cell subtypes, tumor-infiltrated immune cells, and non-immune cells were classified by Seurat and identified by SingleR. Gene sets included cancer stem cells [50], T cells [51], macrophages [52] and B cells [52]. The state transformation direction evaluation of different tumor cell subtypes was performed by velocyto. Lineage trajectory plots were generated using the Monocle R package.

AUC scores were used to further analyze the iron activity of cell clusters, TAM-tumoral, immune, and stromal intercellular communications networks based on potential receptor-ligand interactions. Related regulatory gene networks were constructed using the NicheNetr and CellChat packages. A package enrichplot was used for functional analysis.

### Unsupervised clustering and functional analysis of tumors

Raw count data of RNA sequencing was preprocessed by the edgeR package [53]. R package removebatcheffect was used to remove batch effects of 33 tumors. Identified TAM-communication gene signatures were used as input in the unsupervised classification of TCGA pan-cancer samples. Tumor samples were subsequently classified into different groups based on the gene signature expression pattern. The proportion of TME subtypes among the different tumor types was evaluated by PCA and CIBERSORT.

A target gene regulatory network was constructed by GRNinfer [54] and GVedit (http://portableapps.com/node/38245; version 2.38). GRNinfer gene networks are based on linear programming and determined network structures are consistent, improving results reliability. All of the networks for the dataset are represented as follows: J = (X′ − A) U^∧1^V^T^ = YV = J + YV^T^, where J = (J_ij_)_m×m_ = ∂f(x)/∂x is a connectivity matrix, X = (x(t_1_), …, x (t_m_)) and all n × m matrices with ${\text{x}^{{\prime}}_{\text{i}}}$ (t_j_) = [x_i_(t_j+1_) − x_i_(t_j_)]/[t_j+1_ − t_j_] for i = 1,…,n; j = 1,…m. X(t) = (x_1_(t),…,x_n_(t)^T^∑R^n^, a = (a_1_…,a_n_)^T^∑R^n^, xi(t) is the expression level of gene i at time instance t, y = (y_ij_) is a n × n matrix, U is a unitary m × n matrix of eigenvectors, and ^∧^ = diag (e_1_,…, e_n_) is a diagonal n × n matrix. The parameters were set at λ = 0.0 and threshold = 1 × 10^–9^. The stemness score of each sample [55] was correlated with gene expression levels to evaluate the connection between tumor stemness and hub genes. The estimate scores were interrogated to determine the correlation between gene expression and TME.

### Cell culture

Mouse RAW264.7 macrophages and the metastatic B16-F10 mouse melanoma cell line were obtained from the American Type Culture Collection and were cultured as described previously [56, 57]. Human Bone Marrow (BM) samples were required by aspiration from donors at The First Affiliated Hospital of Chongqing Medical University and primary human BM cells were separated from BM, in detail, BM cells that were collected by density gradient centrifugation from BM were first cultured in α-MEM supplemented with 10% FBS [58], next, an auto MACS Pro Separator (Miltenyi Biotec, Bergisch Gladbach, Germany) were used to purified the CD14-positive BMCs, then, the cells were cultured with human hM-CSF at concentration of 25 ng/ml (R&D Systems) for 3 days to induce macrophage differentiation [59]. As for co-culture system, B16 cells and humanized melanoma cells A-375 were seeded in the lower chamber of a 0.4-μm pore, 24-well transwell system (Corning, Glendale, AZ), Raw264.7 cells and Human Bone Marrow-derived macrophages were added to the upper chamber for the co-culture condition.

### Si-Fli1 RNA interference

Cells at a proper density were incubated for 24 h. FLI1 siRNA (25 nM, Ribobio, Guangzhou, China) was transfected with OPTI-MEM (31985070, ThermoFisher Scientific, Waltham, MA) according to the manufacturer’s instructions. The supernatants were then removed and replaced with fresh medium 24 h later. Cells were sequentially harvested for 72 h for further experiments. The siRNA sequences were as follows:

- Fli-1-specific siRNA (Mouse): 5′-CCCUCCGAUACUACUAUGATT-3′ and
- ‘Non-sense’ (Control): 5′-UUCUCCGAACGUGUCACGUTT-3′.
- FLI-1-specific siRNA (Human): 5′-AUUCAUGUGGUCAUACUCCCG-3′ and
- ‘Non-sense’ (Control): 5′-GGAGUAUGACCACAUGAAUGG-3′.

### Cell viability assay

According to the manufacturer’s protocols (Beyotime, China), B16 cells (1 × 10^4^) were seeded and cultured in 96-well plates with the medium from TAMs for 48 h. The medium was removed and CCK-8 solution was added to each well, followed by incubation at 37 °C in 5% CO_2_ for 4 h. The absorbance was measured in 450 nm.

### Scratch-wound and transwell experiment

Confluent melanoma cells were scratched using a 200 µL pipette tip, the plates were washed and photographed at different times, and the wound area was calculated using an inverted microscope (Olympus, Japan). The transwell system was used for the cell invasion assay. Melanoma cells were seeded into the upper chamber which was coated with an extracellular matrix (BD Biosciences, San Jose, CA). The lower chamber was filled with macrophages and following incubation, the cells under the filter were fixed and stained with a crystal violet solution. The number of cells was counted using a phase-contrast microscope (Olympus, Japan).

### Patients

Melanoma tissues were surgically resected from 23 patients at The First Affiliated Hospital of Chongqing Medical University from January 2020 to October 2020. The inclusion criteria were the pathological diagnosis of melanoma and underwent skin transplantation, while exclusion criteria were patients with recurrence, metastasis, cases with incomplete clinical data, and those with an unknown diagnosis. Tissues of adjacent skin (n = 23) were collected from the same patients and rapidly stored in liquid nitrogen for further experiments. Patients in this study signed an informed consent. This study was approved by the Ethics Committee of Chongqing Medical University (Approval Number: 2020-155) and conducted in accordance with the Declaration of Helsinki.

### qRT-PCR

RNA was isolated from cultured melanoma cells, TAMs, and human tissues using the UNIQ-10 column RNA Extraction Kit (Sangon Biotech, China) according to the manufacturer’s instructions. Reverse transcription was then conducted using the RR047 cDNA synthesis kit (TaKaRa, China). qRT-PCR was performed in a 7500 Real-Time PCR System (Applied Biosystems, Foster City, CA) using the 2× Power SYBR® Green PCR Master Mix (Invitrogen, USA). Gene expression levels were normalized to GAPDH expression. The primer sequences are as follows:

- Fli-1-F (Mouse) (5′-AGCCACATCCAACAGAGA-3′),
- Fli-1-R (Mouse) (5′-AGAGTCCAGAGTATCCAGTAA-3′),
- Ccl3-F (Mouse) (5′-GACCAAGAGAAACCGG-CAGA-3′),
- Ccl3-R (Mouse) (5′-TCTGCCGGTTTCTCTTGGTC-3′),
- Ccl13-F (Mouse) (5′-TTGAGGCTGAGCCAAA GACC3′),
- Ccl13-R (Mouse) (5′-TGGTAGTGAATATCACAG CCCG-3′),
- Ptprc-F (Mouse) (5′-ATGATGGCAGGGTCCTTCATGT-3′),
- Ptprc-R (Mouse) (5′-AGCTTCCTCAGCTAATGATTGGACA-3′),
- Cd28-F (Mouse) (5′-GCTGCTCTTGGCTCTCAACTTATT-3′),
- Cd28-R (Mouse) (5′-CCGCATTGTCGTACGCTACA-3′),
- FLI-1-F (Human) (5′-CCAACGAGAGGAGAGTCATCG-3′),
- FLI-1-R (Human) (5′-TTCCGTGTTGTAGAGGGTGGT-3′),
- CCL3-F (Human) (5′-GGCTCTCTGCAACCAGTTCT-3′),
- CCL3-R (Human) (5′-TTCTGGACCCACTCCTCACT-3′),
- CCL13-F (Human) (5′-ACGTCCCATCTACTTGCTGC-3′),
- CCL13-R (Human) (5′-TTCAGGGTGTGAGCTTTCCG-3′),
- PTPRC-F (Human) (5′-ACTCTTGGCATTTGGCTTTGC-3′),
- PTPRC-R (Human) (5′- CTCCAGTGGTTTGTGAGGGG -3′),
- CD28- F (Human) (5′-GCTGCTCTTGGCTCTCAACT-3′), and
- CD28-R (Human) (5′-GGCATAGGGCTGGTAATGCT-3′).

## Supplementary Information

**Additional file 1: Figure S1.** The experiment of the coculturing humanized melanoma cells A-375 with Human Bone Marrow-derived macrophages (A) FLI1 expression and its targets after FLI1 knockdown, under co-culture conditions (B). The protein levels of secreted CCL3 following FLI1 knockdown (C), A-375 cells viability when co-cultured with Human Bone Marrow-derived macrophages; detected by a CCK-8 assay. (D) The invasion of A-375 cells after co-culturing with Human Bone Marrow-derived macrophages, detected by a transwell invasion assay. (E) The migration of A-375 cells after co-culturing with Human Bone Marrow-derived macrophages; detected by a wound healing assay. (F) Immune cells infiltration landscape conducted by Xcell. (G) Relative expression of Fli1 before and after chemotherapy.

## Data Availability

The raw data supporting the conclusions of this article will be made available by the authors, without undue reservation.

## Abbreviations

TAMs: Tumor-associated macrophages
TME: Tumor microenvironment
CPIs: Immune checkpoint inhibitors
HNSC: Head neck squamous cancer
CCGS: Cell communication gene signatures
PCA: Principal component analysis
DNAss: DNA stemness score
RNAss: RNA stemness score
PWM: Position weight matrix
ccRCC: Clear-cell renal cell carcinoma
HLA-G: Human leukocyte antigen-G
