# Supplementary figures and images for: Pan-cancer analysis reveals tumor-associated macrophage communication in the tumor microenvironment
Source: Exp Hematol Oncol. 2021 May 10;10:31. doi: 10.1186/s40164-021-00226-1 (PMC8108336; doi:10.1186/s40164-021-00226-1)

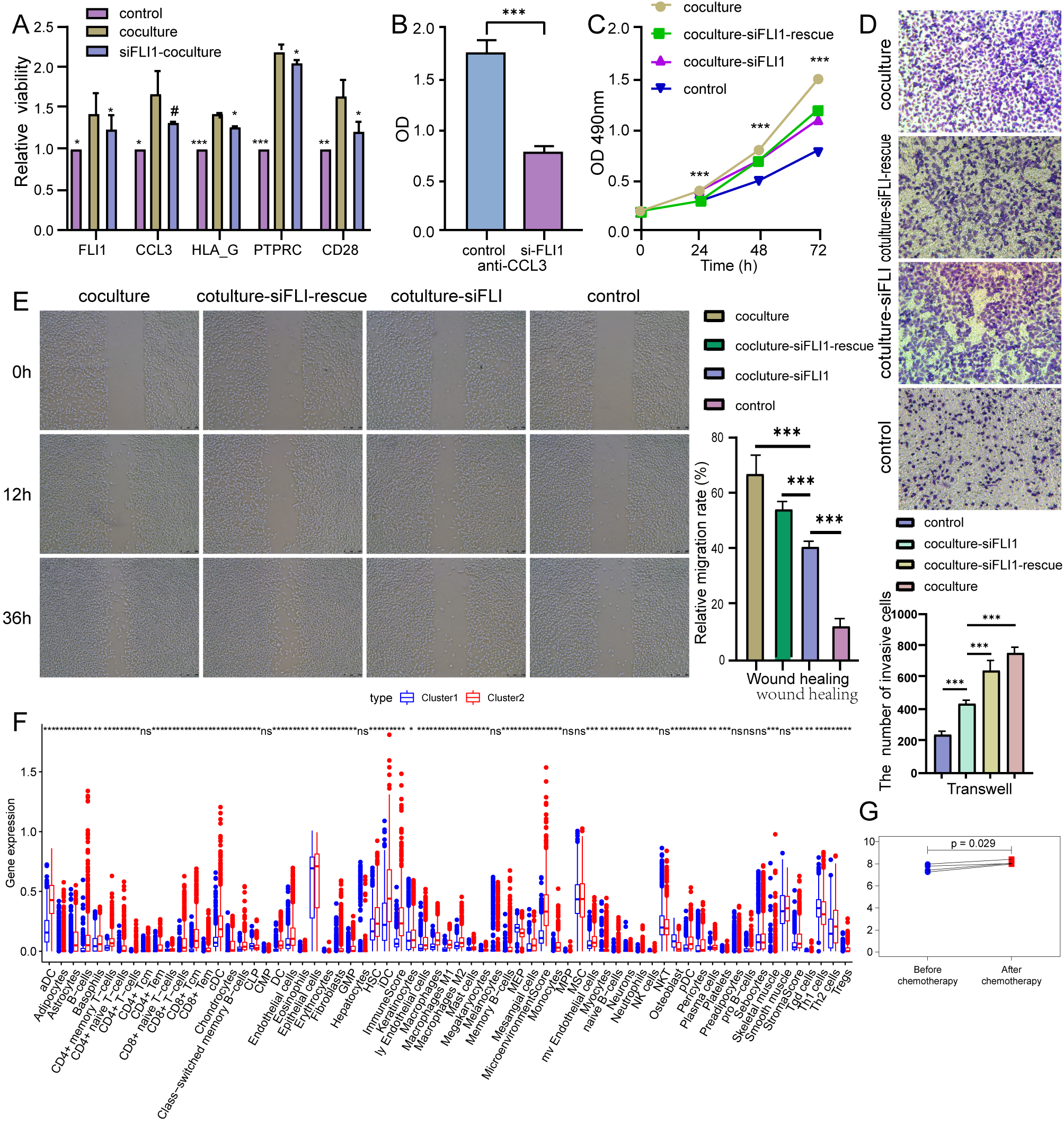

Supplement: Supplementary file 1 — Additional file 1: Figure S1. The experiment of the coculturing humanized melanoma cells A-375 with Human Bone Marrow-derived macrophages (A) FLI1 expression and its targets after FLI1 knockdown, under co-culture conditions (B). The protein levels of secreted CCL3 following FLI1 knockdown (C), A-375 cells viability when co-cultured with Human Bone Marrow-derived macrophages; detected by a CCK-8 assay. (D) The invasion of A-375 cells after co-culturing with Human Bone Marrow-derived macrophages, detected by a transwell invasion assay. (E) The migration of A-375 cells after co-culturing with Human Bone Marrow-derived macrophages; detected by a wound healing assay. (F) Immune cells infiltration landscape conducted by Xcell. (G) Relative expression of Fli1 before and after chemotherapy. [file 40164_2021_226_MOESM1_ESM.tif]
